# Red zones: the true color behind the myth of blue zones geographic longevity

**DOI:** 10.15446/rsap.V27n3.119673

**Published:** 2025-05-01

**Authors:** Jairo Echeverry-Raad, Joachim P. Sturmberg

**Affiliations:** 1 JER: MD. Esp. Pediatría. Facultad de Medicina, Universidad Nacional de Colombia. Fundación Universitaria Juan N. Corpas. International Society for Systems and Complexity Sciences for Health (ISSCSH). Bogotá, Colombia. jecheverryr@unal.edu.co; jairo.echeverry@juanncorpas.edu.co Universidad Nacional de Colombia Facultad de Medicina Universidad Nacional de Colombia Colombia jecheverryr@unal.edu.co jairo.echeverry@juanncorpas.edu.co; 2 JS: MD. Academic GP, MBBS,MFM, FRACGP, Ph.D. School of Medicine and Public Health, Faculty of Health and Medicine, University of Newcastle, Australia. International Society for Systems and Complexity Sciences for Health (ISSCSH). Central Coast Research Institute. Gosford, Australia. jp.sturmberg@gmail.com University of Newcastle Faculty of Health and Medicine University of Newcastle Australia

**Keywords:** Longevity, aging, geographic factors, diabesity, health Behavior *(source: MeSH, NLM)*, Longevidad, envejecimiento, factores geográficos, diabesidad, comportamientos en salud *(fuente: DeCS, BIREME)*

## Abstract

This essay critically analyzes the scientific validity of Ancel Keys' Lipid Hypothesis and the concept of Blue Zones (BZ), both widely accepted as paradigms of longevity and health. The Lipid Hypothesis, derived from the "Seven Countries Study", linked animal-based saturated fat consumption to cardiovascular disease, fostering "lipophobia" and promoting diets rich in carbohydrates and ultra-processed vegetable oils. Paradoxically, these dietary shifts have been associated with a global rise in chronic metabolic diseases such as diabesity. Similarly, the BZ concept, popularized by Dan Buettner and National Geographic, suggested that regions like Sardinia, Okinawa, and Ikaria harbor populations with extreme longevity due to lifestyle factors such as diet, physical activity, and community support. However, the lack of comprehensive global epidemiological studies, biased population selection, and uncontrolled confounding variables compromise these claims. Moreover, recent investigations indicate that many longevity records in BZs may result from clerical errors or fraudulent documentation, especially in regions with unreliable vital records. This paper highlights the emerging evidence that both the low-comorbidity longevity attributed to BZs and the implications of the Lipid Hypothesis are largely based on flawed data. Additionally, it highlights how the food and pharmaceutical industries have leveraged these models to promote the marketing of BZs, excessive medicalization, and high-carbohydrate diets like the Mediterranean diet, exacerbating the current pandemic of chronic diseases.

## RESUMEN

Este ensayo analiza críticamente la validez científica de la hipótesis lipídica de Ancel Keys y el concepto de zonas azules (ZA), ambos ampliamente aceptados como paradigmas de longevidad y salud. La hipótesis lipídica, derivada del "Estudio de los Siete Países", vinculó el consumo de grasas saturadas de origen animal con enfermedades cardiovasculares, fomentando la "lipofobia" y promoviendo dietas ricas en carbohidratos y aceites vegetales ultra-procesados. Paradójicamente, estos cambios dietarios se han asociado con un aumento global de enfermedades metabólicas crónicas como la diabesidad. De manera similar, el concepto de ZA, popularizado por Dan Buettner y National Geographic, sugirió que regiones como Cerdeña, Okinawa e Ikaria albergan poblaciones con longevidad extrema gracias a factores como la dieta, la actividad física y el apoyo comunitario. Sin embargo, la ausencia de estudios epidemiológicos globales, la selección sesgada de poblaciones y la falta de control de variables de confusión comprometen estas afirmaciones. Además, investigaciones recientes indican que muchos de los registros de longevidad en las ZA pueden deberse a errores clericales o documentación fraudulenta, especialmente en regiones con sistemas de registro poco confiables. La evidencia emergente sugiere que tanto la longevidad con baja comorbilidad atribuida a las ZA como las implicaciones de la hipótesis lipídica se basan en datos erróneos. Asimismo, este ensayo destaca cómo las industrias alimentaria y farmacéutica han aprovechado estos modelos para comercializar las ZA, fomentar la medicalización excesiva y promover dietas altas en carbohidratos, como la mediterránea, contribuyendo a la actual pandemia de enfermedades crónicas.

After World War II, the rise of agribusiness prompted epidemiological studies such as the Seven Countries Study, led by Ancel Keys. This study suggested that in certain parts of the world there was a lower incidence of cardiovascular diseases, which led to the birth of the Lipogenic Hypothesis of cardiovascular disease 1. This theory promoted the elimination of saturated fats from the diet, replacing them with vegetable oils and carbohydrates, fostering a widespread fear of animal fats, known as "Lipophobia" 2. This dietary model became solidified in concepts like the Mediterranean Diet and the Food Pyramid, which dominate global nutrition to this day 3.

At the same time, the observation of extreme longevity in certain geographic regions, termed "Blue Zones", generated growing interest in studying the factors that could explain these phenomena. Factors such as diet, physical activity, and a sense of community were highlighted as potential drivers of longevity in these regions 4.

In 2000, Gianni Pes and Michel Poulain coined the term Blue Zone referring to regions like Sardinia, Italy, where the population appeared to enjoy exceptional longevity. This concept was subsequently popularized by Dan Buettner through National Geographic, extended the list to include other areas with remarkable longevity like Okinawa - Japan, and Loma Linda, California, USA 5.

This essay aims to expose the scientific inconsistencies surrounding the Lipid Hypothesis and Blue Zones, arguing that both models are based on flawed methodologies, erroneous and biased data, as well as potential academic fraud. The analysis of these paradigms reveals significant methodological flaws and misleading conclusions, necessitating a reevaluation of their impact on public health policy.

## The paradigmatic fraud of nutrition, longevity, and quality of life 

### The Lipid Hypothesis

Following World War II, the need to boost agribusiness led to a shift in dietary habits. This transition prompted on of the largest epidemiological population health studies in history: the Seven Countries Study, launched in 1956 under the leadership of Ancel Keys 6. This study suggested that certain geographic regions of the world had lower incidences of cardiovascular diseases 1. From this, researchers identified a "healthy" pattern of nutrition and lifestyle in these regions, giving rise to the so-called "Lipid Hypothesis" 7, which eventually led to "Lipophobia" 2. Keys' assertions of lipid being the cause of coronary heart disease though were already challenged in the late 1950s showing that carbohydrate rather than fat consumption were driving obesity and heart disease 8,9.

This theory gained traction through the promotion of specific dietary models like the "Mediterranean Diet," the fitness culture, and the Food Pyramid, which demonized saturated fat while promoting the consumption of Poly Unsaturated Fatty Acids (PUFAs) of heat extracted vegetable oils, plants, seeds, and simple and complex carbohydrate-rich foods, especially fructose, from both natural and processed sources. Today, along with added sugar, these carbohydrates account for between 65% and 75% of the proportion of this non-essential nutrient in the standard global diet, correlating with higher rates of obesity, diabetes, diabesity, and chronic diseases 3,10.

## Longevity and the Blue Zone Concept

At the same time, the observation of exceptional longevity in these areas, including octogenarians, nonagenarians, and even centenarians and supercentenarians, sparked observational studies into potential nutritional factors and lifestyle, as well as the potentially contributing effects of social relationships, biomarkers, and genetic variance. The studies postulated the premise that these factors could be (potentially modifiable) drivers of extreme longevity.

At the turn of the 20th century, the global health perspective shifted beyond the primary focus on morbidity and mortality rates to emphasize the importance of quality of life, spirituality, and dignity, i.e. embracing the need for personalized health 11, beyond the quest for longer 'disease-free' life expectancy or extended life beyond what is expected for each individual according to their context 12.

In 2000, Italian demographer Gianni Pes and Belgian Michel Poulain coined the term "Blue Zone" (BZ) 4,13,14. They, for the prosaic reason, circled with a blue pen the island of Sardinia, Italy, to indicate it as a region of high longevity. From this initial discovery, the concept expanded to include additional regions with similar patterns of longevity and healthy lifestyle.

Later, American explorer and writer Dan Buettner (who is not a scientist), based on propaganda and marketing strategies, popularized the "Blue Zones" concept through National Geographic arbitrarily focusing on five regions with similar characteristics and Northern Latitude 5:

1. Sardinia, Italy -A mountainous region with a high proportion of centenarian men. ~39°N;
2. Ikaria, Greece -With one of the lowest rates of dementia and chronic diseases. ~37°N;
3. Loma Linda, California, USA -Home to a Seventh-day Adventist community, who live 10 years longer than the U.S. average. ~34°N;
4. Okinawa, Japan -Where women have the highest life expectancy in the world. ~26°N; and
5. Nicoya, Costa Rica -A peninsula where its inhabitants have significantly greater longevity than the average. ~10°N.

## The Commercialization of Blue Zones

Under the current business model, Dan Buettner sold his organization, Blue Zones® - BZ®-, to Adventist Health, a network promoting a comprehensive, community-based health approach grounded in the principles of longevity identified in the so-called "BZ®." This multimillion-dollar acquisition, which includes Loma Linda as one of the original BZ, strategically aligned with Adventist Health's values, reinforcing its commitment to a wellness-focused approach to improve community health by optimizing environments and facilitating healthy nutrition, such as plant-based diets, consistent physical activity, a sense of community and purpose, and stress management 15,16. Therefore, the concept of "BZ®," is used to artificially modify the common lifestyle and dietary factors in these areas, theoretically contributing to longevity.

However, the arbitrary selection of the "Blue Zones" is based on anecdotal reports and media appeal rather than a rigorous global epidemiological study that scientifically validate the nexus between extreme longevity with quality of life and its associated socioeconomic factors 13,17. Moreover, the concept of "quality of life" varies culturally and politically, making its generalizability problematic. For instance, Western societies may define "quality of life" through a more hedonistic lens, while Eastern cultures, and other regions, often incorporate spiritual and communal dimensions 18. The lack of solid internal validity to unambiguously define the criteria of "quality of life" or well-being in turn hampers the external applicability of the BZ concept. Loma Linda, where its implementation appears artificially and selectively enforced 5, cannot be regarded as a reliable source in support of this hypothesis.

## A Flawed Health Paradigm?

The BZ model is often presented as an ideal for global longevity and well-being. Outside these zones, the rest of the world-or "Non-Blue Zone", is a "Red Zone" facing the exponential growth in cardiovascular disease, cancer, dementia, diabetes, overweight, obesity, or "diabesity", and all the conditions emerging from these which shorten life expectancy. These conditions now account for 75% of global morbidity and mortality, precisely the diseases that Keys' nutritional model and Blue Zones framework were supposed to prevent 3,10,19.

## Reevaluating the lipid hypothesis and the blue zones paradigm

Today, thanks to dozens of studies and the stubborn demonstration of statistics, we know that the nutritional intervention based on the lipid model proposed by Keys - along with the Blue Zones concept-was not only wrong but fundamentally misguided 19,20.

## Methodological issues in the seven countries study

Critically reviewing the Seven Countries Study 9,19 reveals significant methodological errors by Keys and his team. Whether due to naivety, ignorance, or deliberate bias, they selected populations with a greater predisposition to their desired outcomes, failing to control for critical confounding variables, especially the magnitude of dietary plant, vegetable, and carbohydrate intake-mainly due to a lack of records 21.

It is rumored that Keys began the study with 25 countries but, post hoc, that is, eliminated 18 countries where the statistical correlation between saturated fat consumption and cardiovascular was either nonexistent or inconsistent 22. This data manipulation was reportedly denounced at the time by one of the fathers of population statistics 23, and has since been criticized for its clear logical inconsistencies 24.

## The role of saturated fat and the misclassification of essential nutrients

Contrary to Keys' claims, we now know that saturated animal fat is not only essential for life, survival, reproduction, and physical and mental health, but particularly for the function of the brain and vital organs. In fact, research suggests that our diet should be made up of at least 50% of daily animal fat intake 25.

However, carbohydrates-both natural and processed-have increased sixfold in the contemporary diets compared to 80 years ago. Despite this, they are not considered essential macronutrients in a biochemical, biological, or evolutionary sense. Humans do not require them for survival, yet they now constitute the bulk of our diet 19,20,25.

## Questioning the blue zones narrative

On the other hand, the media phenomenon of the BZ concept raises an intriguing paradox: where reliable and valid vital statistics are available in these regions, the in habitants of these selected geographic areas are characterized by poverty, advanced-age labor, deprivation, and illiteracy-factors that are associated and lower longevity^26^.

There are strong suspicions of deliberate misclassification of individuals as long-lived or centenarian 27, particularly regarding age and age-related diseases. This distortion reinforced Keys' lipogenic model and the Blue Zones narrative, shaping a globally influential "healthy" diet and lifestyle paradigm 18,19,20.

There are serious suspicions that some BZ individuals were falsely -or potentially deliberately-classify as long-lived or centenarian 27. In particular, the "objective" variables used to validate both Keys' lipid model and the Blue Zones framework-such as age records and disease prevalence-may have been inaccurately recorded or deliberately misrepresented, challenging the validity of widely promoted "healthy" dietary and lifestyle recommendations 19,28.

## New evidence challenges the validity of extreme longevity claims

Recently published -and still in preprint 29 - data, have further fuel skepticism regarding the veracity of extreme longevity records as quoted for the BZ population. Specifically, the age documentation of supercentenarians (SC) (people reaching 110 years or more) and semi-su-percentenarians (SSC) (people reaching 105 years) is now under intense scrutiny.

Researcher Saul Newman, with an ingenious question and methodology, investigated official SC and SSC records from various sources, including the Gerontology Research Group (GRG) and the International Database on Longevity (IDL). Applying mixed multivariable regression models, Newman analyzed demographic patterns and their potential correlation with socioeconomic factors, such as poverty rates, incomplete vital records, and crime rates across regions of the U.S., France, Japan, England, and Italy. The results are striking:

- In the United States, the introduction of birth certificates was associated with an 80% decrease in the number of recorded supercentenarians, suggesting a link between the lack of documentation and the appearance of these cases.
- In Italy, poorer regions with lower life expectancy had a higher proportion of supercentenarians, reinforcing the hypothesis that many of these records are due to errors or fraud in registration.
- In France, the ultra-peripheral regions (overseas) and former French colonies were overrepresented in terms of supercentenarians, despite being areas with low life expectancy and little documentation.
- In Japan, the poorest prefectures also showed a higher proportion of centenarians, with patterns similar to those observed in other countries.
- Statistical analysis of birth date patterns revealed an anomalous pattern in birth dates-individuals registered as SCs were more likely to have been born on dates divisible by five, suggesting manipulation or deliberate errors in the records.

## Reassessing the lipid hypothesis and the blue zones narrative: a call for scientific integrity

Evidence is accumulating that many official longevity records may have resulted from administrative errors or potentially outright fraud-with the lack of birth certificates playing a critical role in the "appearance" of these SC cases. This raises serious doubts about the validity of previous studies that relied on these exceptional age records.

Paradoxically, the spurious outcome variable of "extreme longevity" -the key in both the lipid and BZ model- was associated, in multivariable models, to correlate not with superior health and lifestyle choices, but with poverty, low literacy, and the absence of adequate vital records. This revelation topples the house of cards upon which many previous longevity studies were built.

## The blue zones trap and the clerical bias phenomenon

This erroneous or potentially fraudulent classification of longevity in the BZ, may not only extend to the sample of Keys' Seven Countries Study, but also-most likely inadvertently-to other datasets from the mid-20th century, when globally official records of birth and death were far less reliable.

In fact, the entire European cohort of individuals in Keys' Seven Countries Study had been severely compromised during World War II, as churches, town halls, and civil registries were destroyed, leading to massive losses of birth records 30. If true ages could not be properly verified, then the entire premise of identifying long-lived populations based on dietary habits is fundamentally flawed.

In addition to the clear selection and measurement bias in prior diet and longevity studies that underpinned the paradigms of the lipid theory and the Blue Zones, the arguments are further reinforced by the oldest known statistical bias in the history: clerical bias 31. Clerical errors are administrative mistakes (whether naïve, due to ignorance, or deliberate) in the registration, processing, or transcription of information into official documents, with significant consequences 32. These errors typically occur during data handling, such as incorrectly recording dates, names, ages, or other key details. They may also arise for other reasons, such as human oversights or misunderstandings during the documentation process, or deliberately to allow individuals to falsify their age to gain financial benefits, such as early access to pensions or social programs 31,33.

## The broader implications: how clerical errors distort public health data

These issues are particularly relevant in developing nations, where weak registration systems introduce uncertainty into official health insurance, coverage, service delivery, or billing records.

This includes Colombia's prominent registration system known as the "Sistema de Identificación de Potenciales Beneficiarios de Programas Sociales" (SISBÉN) 34, which the Colombian government uses to classify populations to gain access to poverty, vulnerability, and social programs, or, among others, the "Registro Individual de Prestación de Servicios de Salud" (RIPS) 35, which Colombian health companies routinely use for tracking billing and care tracking, including information on diagnoses, treatments, and procedures performed.

Despite these limitations, most graduate-level research in Colombia, South America, continues to rely on these naïve datasets, compromising the validity of their research 36.

The practical impossibility of verifying true longevity through Carbon-14 dating further complicates the issue, allowing for the overestimation and/or underestimation of extreme longevity of individuals or populations.

Failing to address this issue violates the first principle of research-transparency 37. Relying on an unreliable variable-date of birth being open to falsification in official records or being unobtainable 38-calls in question the so-called "gold standard" for validating longevity. Keys' lipid the BZ's nutrition hypothesis stand on spurious foundations-unreliable or unverifiable age-which have artificially shaped global views on health, nutrition, lifestyles, quality of life, and well-being 39.

## The agnotological veil and the global health crisis

**Figure 1:**
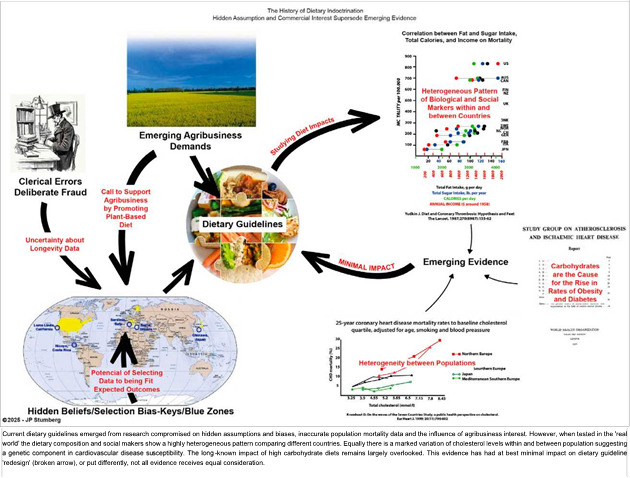
Rich Picture Diagram - Dietary Guidelines

This evidence suggests that the agnotological veil-a term referring to the deliberate or accidental spread of between Historical Assumptions and Scientific Evidence ignorance-is finally being lifted. For decades, scientific knowledge has been distorted and spread deeply within society, influencing mainstream recommendations about human nutrition and lifestyle (Figure 1) 40,41.

The result? A false or spurious nutritional paradigm- perhaps born from best intentions-has ultimately fueled the worst persistent and growing pandemic in history: the pandemic of the non-communicable chronic metabolic disease, obesity, diabetes, or diabesity 19. This will not only make extreme longevity unattainable but rather may barely allow us to reach the life expectancy predicated by our genetic variables and exposure decisions 42.

In the meantime, this failed nutritional model will come at a high cost, leading to the intense medicalization of life, "ad libitum" pharmaceutical consumption, and artificial medical interventions, all driven by a system that prioritizes symptom management over root-cause solutions 43,44.

## What Will It Take for Change?

So, the final question remains: How much more evidence do we need to address our problems in the "Red Zone"?

At what point will we abandon the failed dietary guidelines rooted in flawed data and unverified claims? When will we correct course in our approach to health, nutrition, and longevity?

The time for change is long overdue. Will we take action, or will we continue to let flawed science dictate our future?

The Lipid Hypothesis and Blue Zones paradigm suffer from methodological flaws, biased data selection, and potential academic fraud, misleading public health policies for decades. The Seven Countries Study selectively included data to support a predetermined narrative, demonizing saturated fats and promoting a high-carb diet. However, evidence suggests saturated animal fats are vital for health, while excess carbohydrates contribute to metabolic disorders like obesity and diabetes.

The Blue Zones concept, despite its popularity, lacks scientific rigor. Arbitrary region selection, anecdotal evidence, and age record errors undermine longevity claims. Socioeconomic factors like poverty and illiteracy may have inflated lifespan statistics, and statistical anomalies suggest many supercentenarians were miss recorded.

The commercialization of Blue Zones exacerbates the issue, with corporate interests prioritizing profit over science. Adventist Health's acquisition of Blue Zones® underscores ideological and financial motivations, casting doubt on the objectivity of its recommendations.

The acceptance of these flawed paradigms has contributed to chronic diseases, over-medicalization, and misguided dietary guidelines. A shift toward empirical evidence, transparency, and a true understanding of human physiology is essential. How much longer will flawed paradigms dictate our health decisions before we demand change?♥
